# A Scoring Method to Prioritize Fecal Occult Blood Testing as a First Step in Colorectal Cancer Screening in Resource-Limited Settings

**DOI:** 10.3390/diagnostics13152556

**Published:** 2023-08-01

**Authors:** Linda-Nicoleta Bărbulescu, Virginia-Maria Rădulescu, Stelian-Ștefăniță Mogoantă, Lucian-Florentin Bărbulescu, Constantin Kamal, Mirela Radu, Liana Cismaru

**Affiliations:** 1 Doctoral School, University of Medicine and Pharmacy of Craiova, 200349 Craiova, Romania; 2 Cabinet Medical Dr. Profir I. Mirela SRL, 200145 Craiova, Romania; 3 Department of Medical Informatics and Biostatistics, Faculty of Medicine, University of Medicine and Pharmacy of Craiova, 200349 Craiova, Romania; 4 Department of Automation and Electronics, University of Craiova, 200585 Craiova, Romania; 5 Department of Surgery, University of Medicine and Pharmacy of Craiova, 200349 Craiova, Romania; 6 Department III of Surgery, University Emergency County Hospital, 200642 Craiova, Romania; 7 Department of Computers and Information Technology, University of Craiova, 200585 Craiova, Romania; 8 Department of Family Medicine, University of Medicine and Pharmacy of Craiova, 200349 Craiova, Romania; 9 Department of Pediatrics, University of Medicine and Pharmacy of Craiova, 200349 Craiova, Romania

**Keywords:** body mass index, obesity, diabetes mellitus, colorectal cancer screening, risk factors

## Abstract

This study aims to develop a scoring method that can be used by primary care physicians from remote areas or resource-limited settings to estimate the need for fecal occult blood testing (FOBT) as a first step in colorectal cancer screening. This method relies on several modifiable risk factors that can influence a positive FOBT, an indication of the presence of colorectal polyps, or even colorectal cancer. The scoring method considers, besides the age and gender of the patient, the body mass index (BMI), smoking status, and the diagnoses of diabetes mellitus (type 2 diabetes), dyslipidemia, and hypertension. It does not need any paraclinical exams, which is an advantage when access or material resources are limited. The retrospective study was spread over forty-three months, respectively, from October 2019 to April 2023, and included 112 patients. The score that we designed is a numerical value between 0 and 7. The values between 0 and 3 represent a smaller risk of a positive FOBT (9.68%), values 4 and 5 represent a medium risk (14.75%), while values 6 and 7 represent a greater risk (40%). Using this score, a physician can determine if a patient has a greater risk and recommend it to prioritize taking a FOB test.

## 1. Introduction

Colorectal cancer (CRC) is a burden for patients and society. It can be detected through screening and caught early, meaning greater chances for efficient treatment and survival. The incidence of CRC decreased over the years for people over 50 years but increased for younger people [1]. The incidence of CRC in Romania was 12.7% in 2020, higher in males, according to the Romanian National Institute of Public Health [2]. Mortality by CRC was second to mortality by lung cancer in overall cancer deaths in 2020 [3].

Romania does not have a national colorectal cancer screening. This means that there are no dedicated pathways, no trained personnel, and no cancer awareness campaigns. On top of that, general practitioners did not always have the legal right to recommend fecal occult blood testing. This is the reason why some laboratories refuse to do the FOBT without any cost when recommended by a family doctor. Due to deficient infrastructure, Romania has many isolated or hard-to-reach areas, where access to healthcare facilities is often reduced to a general practitioner (family doctor). Many times, such a doctor is put in the situation of prioritizing patients in performing some analyses due to limited resources. Over time, methods have been developed to detect cancer or to predict the risk of developing cancer [4,5]. In one study, body mass index was correlated with albumin and C-reactive protein to create a newly developed inflammatory-nutrition-related biomarker [6] as an independent prognostic predictor of overall survival in patients with colon cancer. All these risk-prediction models are based on several paraclinical exams, which cannot be conducted in many areas.

Current CRC screening methods used widely are stool-based tests (guaiac-based fecal occult blood test and fecal immunochemical test) and invasive tests (flexible sigmoidoscopy and colonoscopy). These screening methods become diagnostic methods when applied to symptomatic patients. There are many more colorectal screening tests, but most screening guidelines now recommend fecal immunochemical test (FIT) and colonoscopy. Colonoscopy is used as a screening test or to follow up on positive results of an initial non-invasive test [7]. 

FIT has the advantage that does not cross-react with dietary meats. Therefore, there is no need to avoid foods with peroxidase activity [8]. It is a low-cost test. A meta-analysis including 19 qualified studies showed that the overall accuracy of FIT was 95% for the detection of CRC with pooled sensitivity and specificity of approximately 79% and 94%, respectively [9]. One of the limitations of FIT is its low sensitivity for detecting colon polyps [10]. 

Colonoscopy is considered the gold standard for colorectal cancer screening and diagnosis. Multiple case-control and prospective cohort studies have estimated cancer mortality to be 29–68% lower among persons who undergo screening colonoscopy than among those who do not [11]. It has its limitations, being time-consuming and resource consuming. It is expensive and invasive with measurable risk and is not acceptable as an initial test to many participants [7]. It is operator dependent and bowel preparation dependent. It requires access to more complex healthcare facilities than a family doctor’s office.

Although we do not know why it appears, we have now gained much knowledge about colorectal cancer risk factors [12]. And what person can integrate all the information about a patient better than a family doctor? Primary care is ground zero for prevention, screening, and early detection of cancer. A negative fecal occult blood test (FOBT) result at colorectal cancer screening does not necessarily mean that the patient is off the hook if risk factors are involved. Colorectal cancer prevention is linked to colorectal polyp prevention [13]. The family doctor can advise the patients regarding modifiable risk factors and actively engage people in prevention. This part of colorectal cancer risk can be preventable [14,15]. The doctor and patient’s efforts should be aimed at behavior modification. The family doctor can recommend some known protective factors: dietary factors (increasing intake of fruits and vegetables, fiber, resistant starch, folic acid and folate, vitamin B6, calcium and dairy products, vitamin D, magnesium, garlic, fish consumption, coffee intake), physical activity, drugs (aspirin and NSAIDs), and hormone therapy in females [16,17].

Several modifiable risk factors are linked with an increased risk of developing polyps and, eventually, colorectal cancer: obesity, physical activity, diet, gut microbiota, smoking, drinking, and diabetes mellitus (DM). These factors can influence other comorbidities that are most found in patients with DM and have a degree of increasing CRC risk: dyslipidemia (DYSL), and hypertension (HTA) [18,19,20,21]. 

Obesity is known to increase cancer risk. The International Agency for Research on Cancer (IARC) identified a Relative Risk of the highest BMI category evaluated versus normal BMI (95% confidence interval) of 1.3 for colorectal cancer. Relative risks from meta-analyses or pooled analyses were 1.2 to 1.5 for overweight and 1.5 to 1.8 for obesity with respect to cancers of the colon [22].

Diabetes mellitus is an independent risk factor for colorectal cancer. Patients with type II diabetes have a 30–50% higher risk of developing colorectal cancer than non-diabetes persons [23,24]. Available evidence suggests that persons with diabetes mellitus and colorectal cancer may be at increased risk for colorectal cancer recurrence, non-response to chemoradiotherapy treatment, and treatment-related complications [25]. A Mendelian Randomization Analysis suggests that high circulating insulin levels, rather than high glucose levels, can be the main driver of the positive associations found between type 2 diabetes and colorectal cancer in observational studies [26]. On the other hand, numerous studies have proven the protective effect of metformin, a widely used anti-hyperglycemic agent [27,28]. Diabetes and obesity interact mutually: obesity-induced inflammatory factors can impair pancreatic β-cells, while chronic hyperinsulinemia and hyperglycemia in turn lead to visceral adiposity [29].

Metabolic syndrome was associated with an increased risk of early onset colorectal cancer. It is defined as the presence of three or more conditions: obesity (abdominal obesity), hypertension, hyperlipidemia, and hyperglycemia/type 2 diabetes. Compared to individuals without a metabolic comorbid condition, those with one, two, or three or more conditions had a 9% (1.09; 1.00 to 1.17), 12% (1.12; 1.01 to 1.24), and 31% (1.31; 1.13 to 1.51) higher risk of early onset CRC. No associations were observed for 1 or 2 metabolic comorbid conditions and diagnosed CRC at 50–64 [30]. A Japanese analysis found that higher systolic and diastolic blood pressure and stage 2 hypertension are associated with a higher risk for incident CRC, even among those without shared risk factors for CRC [31]. A Taiwanese study found that high triglyceride, high cholesterol levels, and metabolic syndrome were to increase the risk of CRC. In addition, DM patients with a triglyceride level ≥ 150 mg/dL and cholesterol ≥ 180 mg/dL had a 4.118-fold higher risk of CRC as compared with a TG level < 150 mg/dL and cholesterol level < 180 mg/dL, which was a significant difference (95% CI, 1.061–15.975; *p* = 0.0407) [32].

Smoking is another important risk factor for CRC. A meta-analysis that summarizes the evidence from 188 original studies found that compared with never smokers, the pooled RR for CRC was 1.14 (95% confidence interval [CI] 1.10–1.18) for current smokers and 1.17 (95% CI 1.15–1.20) for former smokers [33]. A study that assesses CRC risk by categories of smoking behavior and various levels of genetic risk revealed that a substantial proportion of genetically determined CRC risk could be compensated for by abstinence from smoking [34].

Several predictive models have been developed to improve clinical judgment in patients with abdominal symptoms, and some have included quantitative FITs, but none have been fully validated [35]. 

Physicians can use scores as a colorectal cancer risk-prediction tool in clinical practice when needed. There are several scoring systems for colorectal cancer screening based on the Asian and Polish populations [36,37]. We wanted to propose a scoring method that can be used by primary care physicians from remote areas or resource-limited settings to help skip over FOBT or, on the contrary, to insist on taking preventive steps. It also can be helpful in knowing which modifiable risk factors can be influenced to reduce CRC risk. Due to limited or no access to a screening colorectal test, we developed a tool that does not require spending any money on paraclinical tests.

## 2. Materials and Methods

### 2.1. Study Design and Participants

The original study was an opportunistic colorectal cancer screening pilot program that started in October 2019. It was coordinated by a general practitioner from an urban area and was conducted in agreement with the ethical principles of the Helsinki Declaration and the University Code of Ethics on the proper conduct of research. The ethical approval of this research project was issued by the Ethics and Scientific Deontology Commission of the University of Medicine and Pharmacy, Craiova, Romania (Approval letter 184/30 September 2022). All patients signed informed consent before enrolling in the pilot colorectal cancer screening study.

The study design was described elsewhere [38]. The present retrospective study included 112 patients over 40 years old. They were selected from the original population enrolled in the initial study, between October 2019 and April 2023. We wanted to observe any correlation between a positive FOBT result and modifiable risk factors known for colorectal cancer that was already in patients’ recorded data to develop a method that does not need paraclinical exams.

**Figure 1:**
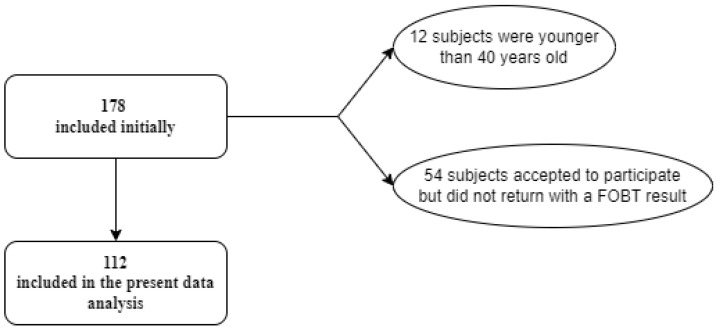
Participant inclusion in the study.

The criteria for excluding patients from the present study are the age under 40 years old and no FOBT result, as presented in Figure 1. A major part of the study was carried out during the COVID-19 pandemic, affecting the number of participants. Following inclusion/exclusion criteria, from 178 subjects we obtained a final cohort of 112 individuals. 

### 2.2. Data Sources

This study is a retrospective analysis of data collected from a pilot screening study.

The patient variables for the present study were obtained from the family doctor’s office database, patients’ charts such as sociodemographic information, height and weight, and comorbidities. Body mass index (BMI) was calculated at enrollment. Dyslipidemia (DYSL), hypertension (HTA), and diabetes mellitus (DM) were identified in patients’ charts, as well as smoking status. FOBT results were collected from the original study. Not all patients with a positive FOBT result had a colonoscopy, but no colorectal cancer was detected in those with one. This is the reason why we did not include it in the present study.

### 2.3. Sociodemographic and Lifestyle Variables

Information about sociodemographic and lifestyle characteristics was collected from the patient’s medical records at enrollment.

Regarding smoking status, participants were asked by their family doctor if they had ever smoked, and whether they currently smoke. Their response was recorded in their patient charts. The participants were classified according to smoking status as non-smokers (patients who never smoked and former smokers) and current smokers (patients who are currently smoking).

### 2.4. Clinical Data

The physical examination included the measurement of anthropometric parameters. Patients with a body mass index (BMI) between 25 kg/m^2^ and 29.99 kg/m^2^ were considered overweight and patients with a body mass index (BMI) ≥ 30 kg/m^2^ were considered obese.

Dyslipidemia (DYSL) was considered when patients were on statin therapy recorded in their medical records from their GP.

Hypertension (HTA) was considered when patients were on antihypertensive treatment recorded in their medical records from their GP.

Diabetes mellitus (DM) was considered when patients were on hypoglycemic treatment recorded in their medical records or from their GP, or their GP received a confirmation letter about their patient diagnosis from other doctors.

## 3. Results

As previously stated, the patients included in the study were those over 40 years old. We chose this starting age for two reasons. First, no patient under 40 years with an FOBT result had comorbidities. Second, although colorectal cancer screening begins at 50 years in many countries, it is recommended to lower this age because of early onset colorectal cancer. We kept the patients over 75 years old because none of them had a previous colorectal cancer screening.

Since the oldest patient included in the study is 88 years old, it was considered that an age range of approximately 48 years would be sufficient, and it would include relevant information for the patients who constituted the focus group. From the statistical analysis of the data recorded in the database, as presented in Table 1, the working hypothesis is correct because a Skewness between −0.5 and +0.5 indicates that the distribution is symmetrical (in the present case, Skewness = −0.27). It makes sense to consider that the population chosen under the previously stated conditions represents a correct hypothesis. Another piece of information reinforcing that the chosen patients are correct would be that Mode is 67. It overlaps with our idea of analyzing what happens to the patients who turn away from the hope of life in the country (Romania).

According to EUROSTAT, the country’s life expectancy in Romania in 2021 was 72.9, below the end age for colorectal cancer screening. Table 2 presents a distribution of life expectancy in Romania’s regions. The study took place in the South-West Oltenia region.

Of 178 individuals completing the first study visit, 112 participants were included in the present data analysis. Table 3 shows the demographic characteristics of the study participants.

Of the 112 unique patients in this study, 106 (94.60%) came from the urban area, the remaining 6 were from the rural area, 51 were males, and 61 were females. The mean age was 65.30 years: 66.00 years for females and 63.69 years for males. Female subjects accounted for 54.46% of the total. Regarding the results of fecal occult blood tests, 20 patients had a positive FOBT result (17.86%), and 92 patients had a negative FOBT result.

We wanted to know the trend of BMI in patients from the study, and that is shown in Table 4.

From the statistical data analysis, the analyzed population’s trend is one of overweight, a fact also emphasized by the three indicators of the center trend from Table 5 (mean = 28.46, median = 27.90, mode = 26.30).

We analyzed the distribution of patients that had a positive FOBT result by BMI and age-range classes as shown in Table 6. Looking at the data, patients with a positive FOBT are mostly male and over the age of 60. Most patients fall into the overweight or obese BMI categories. This suggests that BMI is a crucial factor in analyzing patient conditions. To improve this analysis, it would be helpful to include suggestions on how this data could be used to improve patient care or inform healthcare policy.

**Figure 2:**
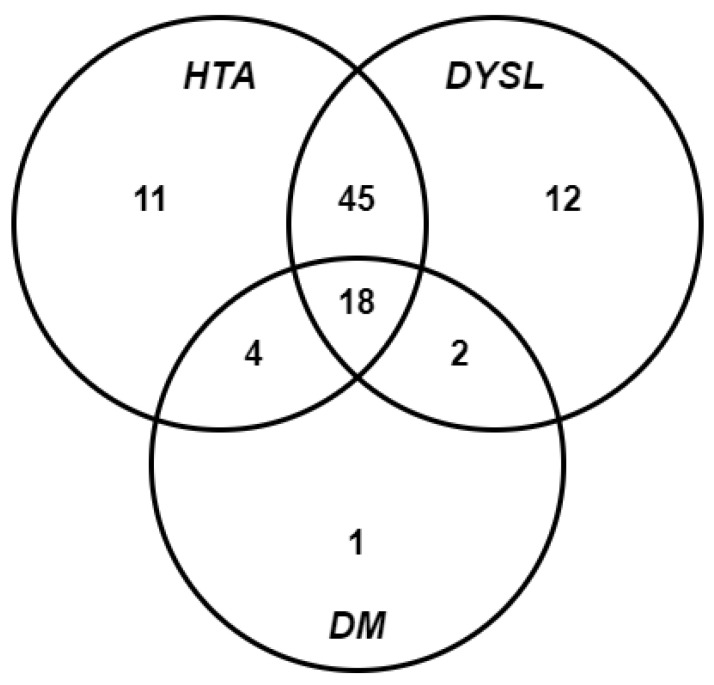
Distribution of type 2 diabetes (DM), dyslipidemia (DYSL) and hypertension (HTA), among the patients from the study group. The numbers represent the patients from each group.

We analyzed the distribution of the three comorbidities we considered, type 2 diabetes (DM), dyslipidemia (DYSL), and hypertension (HTA), among the patients from the study group. We identified 19 patients with none of the conditions and 93 with at least one. Figure 2 illustrates the occurrence of the three risk factors among the patients and their overlap.

Distribution of the patient’s body mass index versus FOBT and DM, DYSL, and HTA is presented in Table 7.

Upon analyzing the data retrieved from Table 7, it became evident that males with a positive FOBT are more susceptible to being affected by at least one of the three comorbidities mentioned in the study.

Additionally, a correlation between FOBT result and patient gender and the number of comorbidities is shown in Table 8.

Based on the observed results and on existing literature, we defined a scoring method that will allocate a value from 0 to 7 to each patient. The score points allocation is presented in Table 9.

By applying the previous score to the cohort of patients available, we obtained the results presented in Table 10.

## 4. Discussion

Romania is one of two EU countries that do not have a population-based colorectal cancer screening. Seeing that the country’s life expectancy was 72.9 years in 2021, near the top end of CRC screening eligibility, maybe it would be a better idea to start screening from a lower age than 50 years old. Another fact that is pleading for a lowering age for starting CRC screening is that red meat consumption is very high in the Romanian population. Traditionally, Romanians consume a lot of pork meat and meat meals. It is known that red meat intake is a colorectal cancer risk factor [18].

We found that 48.21% of patients from this study are overweight and 33.93% are obese. The PREDATORR study found that 31.4% of Romanian adults between the ages of 20 and 79 suffer from obesity and 34.6% are overweight [39].

From the distribution of patients that had a positive FOBT result by BMI and age-range classes, we can observe that only one patient under 50 years old with a positive FOBT result is overweight. Because of the increased risk of early onset colorectal cancer, the family doctor should advise this patient on metabolic risks that are associated with being overweight under these conditions. Most overweight and obese patients with positive FOBT results are over 60 years old.

The proposed scoring method from Table 9 was defined based on both well-known factors that influence a positive FOBT and observed results from the study group. In the literature [40,41], it is recommended to start screening for colorectal cancer in all adults over the age of 50 years with no other known risk factors. Also, the risk of developing colorectal cancer doubles after the age of 60 years. For these reasons, we allocated 0 points for patients under 50 years, 1 point for patients between 50 and 59 years, and 2 points for patients over 60 years.

The gender of the patients is also considered relevant in determining the risk of developing colorectal cancer in the way that males have an increased risk compared to females [42]. For this reason, we considered 1 point for males and 0 points for females.

Because smoking is an important risk factor, we allocated 1 point to the patients who are currently smoking and 0 points to the ones that never smoked or quit smoking.

The body mass index is also considered an important risk factor in developing colorectal cancer [22]. This is why we allocated 1 point for patients with a BMI over 30 and 0 points for patients with BMI under 25. For the patients with BMI between 25 and 30, we analyzed the data from the study and other results from the literature [36,37] and decided to allocate 1 point for females and 0 points for males.

Of the three considered comorbidities, type 2 diabetes, dyslipidemia, and hypertension, the first two are known strong risk factors, while the third is considered an average risk factor. We also observed, by analyzing the data from Figure 2 and Table 8, that a positive FOBT result is more likely for patients that have two or all three conditions. This is why we allocated 0 points for patients with none of the above conditions, 1 point for patients with only one condition, and 2 points for patients with at least two of the conditions.

The proposed scoring method offers a numerical value between 0 and 7. However, because scores between 0 and 3 can be obtained only based on the age and gender of the patient, and those are unmodifiable risk factors, we considered them as part of the same group. Additionally, since our dataset was limited, we did not obtain a maximum score for a patient, and this is why we considered all values above six in the same group. We thus obtained a risk estimation for a positive FOBT of 9.68% for a score between 0 and 3, 10% for a score of 4, 17.07% for a score of 5, and 40% for a score greater or equal to 6.

Table 10 represents a synthesis of the data collected according to the score. As can be seen, there is a high correlation between the proposed score and the determination of positive FOB patients. Moreover, the determination trend is an increasing one.

For the two sets of values, the Student test was applied, and the following values were obtained: the t-value is −3.49663, and the *p*-value is 0.00644. The value obtained for the *p*-value indicates that we obtained a significant result; it is known that the limit for the *p*-value is *p* < 0.05. All this entitles us to consider the method proposed in the study as a correct method, which can lead to good results.

### Study Limitations

The pilot study was conducted in a single center in an urban area. The colorectal cancer screening was an opportunistic one, and most of it was conducted during the COVID-19 pandemic. This explains in part the low number of patients enrolled in the study and the postponed results. Abdominal obesity was not considered because it was not in all patient charts. Very few patients admit to alcohol consumption. It would be important in the future to investigate a scoring method that includes abdominal obesity and alcohol consumption.

## 5. Conclusions

In this article, we developed a scoring method that assesses the risk of a positive FOB test for people over 40 years old. We obtained a risk value that varies from 9.68% to 40%. Based on the computed score, a physician can recommend a patient to prioritize or not having a FOB test completed. This is extremely useful in areas where access to medical resources associated with FOB testing is limited.

This study reveals that overweight patients represent 48.21% of the total cohort and the obese patients represents 33.93% of the studied group. If overweight or obese patients reduce their BMI, they can reduce the score, thus reducing the risk.

The family doctor could prioritize screening patients that have multiple comorbidities because they are more prone to develop multiple pathologies that have common risk factors. In our study, 83.03% of people had at least one comorbidity. If patients can treat their afflictions, the score is reduced.

## Figures and Tables

**Table 1:** Patients age analysis.

Patients Age Analysis	
Mean	65.3
Median	66.5
Mode	67
Standard Deviation	11
Skewness	−0.27
Age Range	48
Youngest patient	40
Oldest patient	88
Count	112
Confidence Level (95.0%)	2.07

**Table 2:** Life expectancy by regions in Romania in 2021.

Region	Life Expectancy (Years)
North-West	72.8
Center	73.3
North-East	72.0
South-East	72.1
South-Muntenia	72.4
Bucharest-Ilfov	73.9
South-West Oltenia	73.4
West	72.5

**Table 3:** Characteristics of the cohort of 112 subjects.

	Patients *n* (%)
Total	112 (100%)
Gender	
- M ^1^	51 (45.54%)
- F ^2^	61(54.46%)
Demographic	
- Urban	106 (94.64%)
- Rural	6 (5.36%)
Mean Age	
- M	64
- F	66.36
Median Age	
- M	66
- F	67
Mode of Age	
- M	55
- F	67
Age-range classes	
- 40–49	8 (7.14%)
- 50–59	26 (23.21%)
- 60–69	38 (33.93%)
- over 70	40 (35.72%)
FOBT ^3^ results	
- Positive	20 (17.86%)
- Negative	92 (82.14%)
Diabetes mellitus (DM)	
- M	11 (44.00%)
- F	14 (56.00%)
Hypertension (HTA)	
- M	34 (43.59%)
- F	44 (56.41%)
Dyslipidemia (DYSL)	
- M	37 (48.05%)
- F	40 (51.95%)
Smoking	
- M	11 (55.00%)
- F	9 (45.00%)

^1^ Males. ^2^ Females. ^3^ Fecal Occult Blood Test.

**Table 4:** Distribution of patients, body mass index versus FOBTs.

BMI ^1^\FOBT ^2^	*n*	*n* (%)	FOBT Positive	FOBT Positive (%)	FOBT Negative	FOBT Negative (%)
Under 18.50	1	0.89	0	0	1	0.89
18.50–24.99	19	16.96	4	3.57	15	13.39
25.00–29.99	54	48.21	8	7.14	46	41.07
30.00–34.99	30	26.79	6	5.36	24	21.43
35.00–39.99	5	4.46	0	0	5	4.46
Over 40	3	2.68	2	1.79	1	0.89

^1^ Body Mass Index. ^2^ Fecal Occult Blood Test.

**Table 5:** Patient BMI analysis.

Patients BMI Analysis	
Mean	28.46
Median	27.90
Mode	26.30
Standard Deviation	4.66
Skewness	0.31
BIM Range	23.60
Minimum BIM	17.60
Maximum BIM	41.20
Count	112
Confidence Level (95.0%)	0.87

**Table 6:** Distribution of patients’ body mass index (BMI) by age-range classes.

Age Range	BMI									
	18.50–24.99		25.00–29.99		30.00–34.99		35.00–39.99		Over 40	
	F	M	F	M	F	M	F	M	F	M
40–49				1						
50–59	1			1						1
60–69	1	1	2	1	1	4				
over 70		1	3			1			1	

**Table 7:** Distribution of patients, body mass index versus FOB tests and DM, DYSL, and HTA.

BMI\FOB	Diabetes Mellitus				Dyslipidemia				Hypertension			
	FOB Positive		FOB Negative		FOB Positive		FOB Negative		FOB Positive		FOB Negative	
	M	F	M	F	M	F	M	F	M	F	M	F
Under 18.50	0	0	0	0	0	0	0	0	0	0	0	1
18.50–24.99	1	0	1	2	2	0	2	6	2	1	3	7
25.00–29.99	0	1	3	4	2	5	17	13	2	4	15	13
30.00–34.99	3	0	3	4	5	1	6	10	3	1	6	12
35.00–39.99	0	0	0	2	0	0	1	4	0	0	1	4
Over 40	0	1	0	0	1	1	1	0	1	1	1	0

**Table 8:** Correlation between FOBT result and patient gender and the number of comorbidities.

DM, DYSL, HTA	None		1		2		3	
	FOB		FOB		FOB		FOB	
Gender	POZ	NEG	POZ	NEG	POZ	NEG	POZ	NEG
F	1	9	2	14	4	19	2	10
M	0	9	2	6	7	21	2	4

**Table 9:** Score points allocation for computing the risk of a positive FOBT.

**Risk Factor**	**Category**	**Point**
Age (years)	40–49	0
	50–59	1
	Over 60	2
Gender	Female	0
	Male	1
Smoking	Not smoking currently	0
	Current smoker	1
Body mass index (kg/m^2^)	<25	0
	25–29.99	Male 0 Female 1
	≥30	1
Other condition	No other condition	0
	One other condition (HTA, DM, DYSL)	1
	Two or all other conditions (HTA, DM, DYSL)	2

**Table 10:** Scoring results.

**FOB\Score**	**0–3**	**4**	**5**	**≥6**
POZ (*n*)	3	2	7	8
NEG (*n*)	28	18	34	12
POZ (%)	9.68%	10.00%	17.07%	40.00%
NEG (%)	90.32%	90.00%	82.93%	60.00%

## Data Availability

The data presented in this study are available on request from the corresponding author.
